# Estimation of body segmental orientation for prosthetic gait using a nonlinear autoregressive neural network with exogenous inputs

**DOI:** 10.1007/s13246-023-01332-6

**Published:** 2023-10-23

**Authors:** Lai Kuan Tham, Mouaz Al Kouzbary, Hamza Al Kouzbary, Jingjing Liu, Noor Azuan Abu Osman

**Affiliations:** 1 Center for Applied Biomechanics, Department of Biomedical Engineering, Faculty of Engineering, Universiti Malaya, Kuala Lumpur, 50603 Malaysia; 2 The Chancellery, Universiti Tenaga Nasional, Kajang, 43000 Malaysia

**Keywords:** Inertial sensors, Prosthetic gait, NARX network, Artificial neural network, Orientation estimation, Validation

## Abstract

Assessment of the prosthetic gait is an important clinical approach to evaluate the quality and functionality of the prescribed lower limb prosthesis as well as to monitor rehabilitation progresses following limb amputation. Limited access to quantitative assessment tools generally affects the repeatability and consistency of prosthetic gait assessments in clinical practice. The rapidly developing wearable technology industry provides an alternative to objectively quantify prosthetic gait in the unconstrained environment. This study employs a neural network-based model in estimating three-dimensional body segmental orientation of the lower limb amputees during gait. Using a wearable system with inertial sensors attached to the lower limb segments, thirteen individuals with lower limb amputation performed two-minute walk tests on a robotic foot and a passive foot. The proposed model replicates features of a complementary filter to estimate drift free three-dimensional orientation of the intact and prosthetic limbs. The results indicate minimal estimation biases and high correlation, validating the ability of the proposed model to reproduce the properties of a complementary filter while avoiding the drawbacks, most notably in the transverse plane due to gravitational acceleration and magnetic disturbance. Results of this study also demonstrates the capability of the well-trained model to accurately estimate segmental orientation, regardless of amputation level, in different types of locomotion task.

## Introduction

Lower limb amputation, the final clinical option for pain and infection control [1], is both life-saving and life-changing. Following the loss of major muscle groups, the gait of lower limb amputees is frequently reported to be different from the able-bodied gait [2–5]. Prosthetic gait can be affected by numerous factors such as socket fit, prosthetic alignment, and the efficacy of prosthetic components [6]. Gait analysis is commonly used to assess the degree of deviation of prosthetic gait as the feedback to prosthesis prescriptions [7]. It is also important for the evaluations of rehabilitation and therapy outcomes, mobility, and fall risk [8]. In prosthetic biomechanics, gait analysis provides useful information in the development of active prostheses and prosthetic components.

Visual observation is the most common and accessible tool for in-clinics prosthetic gait analysis. In order to improve the reliability and sensitivity of the assessments, different scales are normally used to categorize the outcome of observational gait analysis [9–11]. The outcome measure is, however, subjective and qualitative with no guidelines on the selection of relevant assessment scales. Furthermore, gait abnormalities that occur rapidly are hardly noticeable through naked eyes, thus reducing the sensitivity of prosthetic gait assessment [9].

Quantitative gait analysis which involves optical motion capture system provides an objective means to prosthetic gait evaluation. Detailed analysis of body segment and joint movements allows accurate characterization of motion, even with subtle gait deviations, providing systematic documentation to the assessment of an amputee’s gait and rehabilitation progress monitoring [8]. Nevertheless, the availability of motion capture system as part of the clinical settings is often limited by the high setup cost. Moreover, operation of the sophisticated equipment requires skilled personnel with measurements of gait assessment being restricted in a confined capture volume [12–15].

Recent advances in wearable technology have opened up new perspective for prosthetic gait assessment with fewer constraints. In contrast to the optical motion capture system, the ambulatory system consisting of miniature inertial measurement units (IMUs) is portable with simple setup procedures [16]. Such system is capable of providing continuous measurement of human movements beyond the constraints associated with spaces and environments [13, 16].

Accelerometer which measures linear acceleration along its sensitive axis is the most common inertial sensor used in ambulatory gait analysis, thanks to its robustness, durability, low cost and low power consumption [14, 17]. Notwithstanding the special features, high frequency movements negatively affect the accuracy of accelerometer with dynamic errors produced in the mechanical structures of the sensor [17]. Gyroscope which measures angular velocity about an axis is frequently used alongside the accelerometer to compensate the aforementioned limitation. The two sensors are complementary, providing good estimates of orientation by combining the advantages of both sensors at varying movement frequencies. Nevertheless, the accelerometer is also affected by gravity where the existing gravitational acceleration vector confounds dynamic acceleration measured along the vertical axis, restraining measurement to two-dimensional (2D) [17–19].

Accurate measurement in three-dimensional (3D) can be achieved with an additional reference axis to the wearable system. In this regard, magnetometer which detects the local magnetic north is most commonly used to provide a supplementary reference axis [20–22]. The deployment of accelerometer, gyroscope, and magnetometer enables detailed analysis of 3D human gait in terms of kinematics and spatiotemporal parameters through sensor fusion algorithms such as the Kalman filters that extract special features from each sensor [21–24]. In recent years, IMU comprises the accelerometer, gyroscope, and magnetometer has gained interest as the leading means of wearable technologies in clinical applications [25]. Unfortunately, the sensing ability of magnetometer is distorted with the presence of ferromagnetic interference, leading to significant errors in orientation measurements, notably in the heading direction [22, 26]. The issue is ever present in clinical settings which comprise mainly of ferromagnetic materials and magnetic field-based devices. Where characterizing local magnetic field *a priori* in uncontrolled environments can be laborious, compensation of ferromagnetic interference thus remains the major challenge of 3D ambulatory gait analysis in clinical applications [22, 26, 27].

The current study presented an alternative to compute the kinematics of prosthetic gait using a neural network model which requires only the gyroscope data. Presented with chaotic features in nature, human gait dynamics is frequently analyzed through the nonlinear signal processing methods [28–30]. The nonlinear autoregressive neural network model with exogenous inputs (NARX) is an important class of system identification and prediction for nonlinear time series data, e.g., physiological signals [31, 32]. The approach demonstrated outstanding performance in extracting 3D kinematics of the knee joint for long-distance walking [33] and the control of lower limb exoskeleton robot [34, 35], proving its feasibility as a joint angle measurement tool. The aim of current study was to validate a NARX neural network model in the estimation of prosthetic gait kinematics using only the gyroscope signals. Secondly, the study aimed to show the ability of the NARX model, which was trained using healthy data, in extracting segment orientation of prosthetic gait including complex movement such as turns.

## Method

### Wearable system

**Fig. 1:**
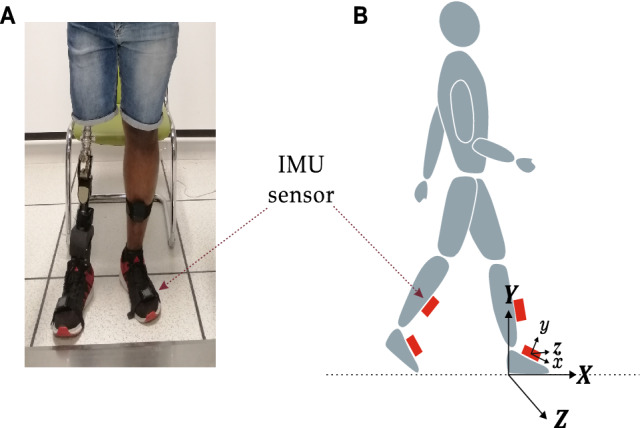
Illustration of the measurement system. **(A)** A subject fitted with inertial sensors on the shank, pylon, foot and artificial foot. **(B)** Definition of the sensor technical frame(*xyz*) and the foot anatomical frame (*XYZ*)

Four IMUs (OPAL, APDM Inc., Portland, USA) attached bilaterally with elastic straps to the shank or pylon and foot (Fig. 1), were used to record gait signals in this study. Each sensor unit consisted of a 3D accelerometer (±11 g), a 3D gyroscope ($\pm 200^\circ$ /s), a 3D magnetometer (±8 Gauss), a memory unit and a wireless transmitter. All sensor units were connected through a wireless synchronization system at sampling frequency 128 Hz. Data were streamed real-time to a computer and stored for analysis.

The IMUs which measured data in the technical frames were aligned to the body segment anatomical frames trough a calibration procedure. The subjects were first requested to stand upright for 10 s and the mean acceleration vector was used to align the *y*-axis to the superior-inferior *Y*-axis (i.e., gravity), pointing upward. The *z*-axis was then aligned to the mediolateral *Z*-axis (i.e., pointing to the right of the subject) of each segment by optimizing the angular velocity vector in the sagittal plane. Finally, the anterior-posterior *X*-axis (i.e., pointing forward) of each segment was obtained as the cross product of the *Y*- and *Z*-axes (Fig. 1). All data in this study are reported in the body anatomical frame unless otherwise specified.

### Subjects and measurement protocol

Thirteen subjects with transtibial and transfemoral prostheses aged between 24 and 69 were enrolled in this study (Table 1). All subjects were experienced prosthetic users of minimum 12 months experience. Individuals who presented ulcer, swelling, sore, or pain on the stump were excluded from the measurement trials.

All subjects performed a two-minute walk test (2MWT) at self-selected speed, with unrestricted U-turns between straight walking [36, 37]. First, each subject walked along a $1 \times 2$m (width $\times$ length) walkway (with support bars for safety consideration) using the originally prescribed prosthetic foot (i.e., passive foot). Then, walking trials were repeated with a robotic foot (RoMicP^®^, Bioapps, Kuala Lumpur, Malaysia) fitted to the prosthesis. Note that the subjects were not trained on using the robotic foot before the second walking trials. All fitting and alignment procedures were performed by professional prosthetists.

**Table 1:** Subject demographic. The age, body mass, and height are presented as mean $\pm$ standard deviation (minimum, maximum)

Gender	13 male
Age (years)	55.23 ± 12.13 (24, 69)
Body mass (kg)	82.31 ± 18.81 (64, 120)
Height (cm)	177.77 ± 5.39 (172, 189)
Amputation side	6 left, 6 right, 1 bilateral
Level of amputation	10 below knee, 3 above knee

### Reference Segment Orientation

The reference orientation of body segment was estimated based on a complementary filter (CF) due to its simple structure and robustness in multi-source data fusion [38, 39]. In the present study, the CF fused data from the accelerometer, gyroscope, and magnetometer. Considering rotation in one-dimension, e.g., the sagittal plane, the acceleration vector (*a*) measured by the accelerometer can be represented as:

1 $$\begin{aligned} a = [a_X \quad a_Y \quad a_Z] \end{aligned}$$

where *X*, *Y*, and *Z* denote the axes of the body anatomical frame defined in the Wearable System Section. Using the acceleration vector, angular position of body segment ($p_{acc}(t)$) can be measured by the accelerometer as:

2 $$\begin{aligned} p_{acc}(t) = tan^{-1}\left( \frac{a_X}{a_Y}\right) \end{aligned}$$

Gyroscope is also capable of estimating angular position ($p_{gyro}(t)$) through a single time integration of angular velocity ($\nu$):

3 $$\begin{aligned} p_{gyro}(t) = \int _0^t \nu _Z(\tau ) \, \textrm{d}\tau \end{aligned}$$

where $\nu _Z(.)$ denotes the angular velocity in the *Z*-axis.

Outputs of the accelerometer and gyroscope, however, are subjected to high frequency and low frequency noises or drift, respectively. Therefore, the CF fused signals of the accelerometer and gyroscope by eliminating drift errors, which can be represented in the Laplace form as:

4 $$\begin{aligned} \begin{aligned} P_{acc\_gyro}(S)&= \frac{k}{S+k}P_{acc}(S) + \frac{S}{S+k}P_{gyro}(S) \\&= \frac{k}{S+k}P_{acc}(S) + \frac{1}{S+k}N_{gyro}(S) \end{aligned} \end{aligned}$$

where $k/(S+k)$ and $S/(S+k)$ are a low pass and a high pass filter, respectively. *k* is the cut off frequency of the filter. $p_{gyro}(t)$ and $\nu _{gyro}(t)$ are related by $N_{gyro}(S) = SP_{gyro}(S)$ in the *S*-domain. In this study, the CF in Eq. 4 provided estimation of segment orientation in the sagittal and frontal planes.

The existence of gravity negatively affects accelerometer signal in the transverse plane, as the rotation around the superior-inferior axis becomes indistinguishable from the gravitational acceleration vector. In this context, the magnetometer was used as a substitute of accelerometer. The magnetic field (*m*) measured by the magnetometer is:

5 $$\begin{aligned} m = [m_X \quad m_Y \quad m_Z] \end{aligned}$$

where *X*, *Y*, and *Z* denote the axes of coordinate system similar to the accelerometer. Rotation or angular position can then be measured by the magnetometer as:

6 $$\begin{aligned} p_{mag}(t) = tan^{-1}\left( \frac{m_Z}{m_X}\right) - p_0 \end{aligned}$$

where $p_0$ denotes the initial state of the magnetometer with no rotations. Thus, the CF implemented in the transverse plane can be represented as:

7 $$\begin{aligned} P_{mag\_gyro}(S) = \frac{k}{S+k}P_{mag}(S) + \frac{S}{S+k}P_{gyro}(S) \end{aligned}$$

In general, the filters in Eq. 4 and Eq. 7 can be combined to the CF estimating 3D orientation as:

8 $$\begin{aligned} P(S) = \frac{k}{S+k}\left[ \,\alpha P_{acc}(S) + (1-\alpha )P_{mag}(S)\,\right] + \frac{S}{S+k}P_{gyro}(S) \end{aligned}$$

with $\alpha$ being a weight parameter between 0 and 1. This study specifically focused on two cases, $\alpha = 0$ for the sagittal and frontal planes (i.e., Eq. 4) and $\alpha = 1$ for the transverse plane (i.e., Eq. 7).

### Orientation estimation based on NARX

**Fig. 2:**
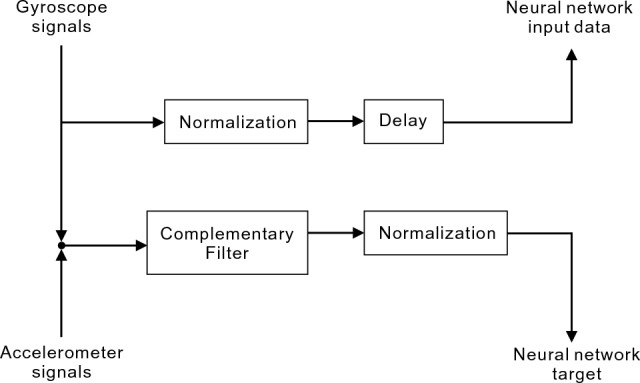
Flowchart for the NARX neural network training. The NARX model was trained with the gyroscope signals of the sagittal plane as input and the orientation of the sagittal plane computed from the combination of gyroscope and accelerometer signals using the CF as the target output of the network

Segment orientation was computed based on the NARX neural network following the method presented in Tham *et. al.* [33]. As illustrated in Fig. 2, training of the NARX model involved IMU signals obtained from healthy gait. Gait signals were first collected from a healthy subject (28 years old, 78 kg, 1.67 m) performing level-ground walking on a treadmill at self-selected speed with the same setup of wearable system as in the Wearable System Section. Fusing acceleration and angular velocity in the sagittal plane, the foot orientation was estimated by the CF described in the Reference Segment Orientation Section and selected as the target output to train the NARX model. On the other hand, angular velocity of the foot, measured by the gyroscope in the sagittal plane, was used as the input in training. Training and testing of the NARX model were then completed using the approach described in Tham *et. al.* [33]. The model was trained using the Bayesian Regularization (BR) algorithm and the training hyperparameters are documented in Table 2.

**Table 2:** Training hyperparameters of the BR algorithm

Maximum number of epochs	75000
Performance goal	0
Marquardt adjustment parameter (mu)	0.005
Decrease factor for mu	0.4
Increase factor for mu	10
Maximum value for mu	$10^{10}$
Maximum validation failure	infinite
Minimum performance gradient	$10^{-18}$

Estimation of segment rotation using the NARX neural network required only the angular velocity signals measured by the 3D gyroscope during gait trials. Structure of the NARX neural network used in this study was similar to the one proposed in Tham *et. al.* [33]. Normalized angular velocity and the recurrent output of angular position constituted the input parameters. A hidden layer with weight distribution established in the network training process comprised three processing neurons that produced output parameters to the output layer. Denormalized output parameters represented 3D segment orientation as flexion and extension in the *Z*-axis, abduction and adduction in the *X*-axis, and internal and external rotation in the *Y*-axis.

### Validation

Estimation errors of segment orientation were computed as the subtraction of 3D orientation estimated by the CF (i.e., reference method) from the NARX model for each time sample (Eq. 9). To assess the performance of the proposed method, the intra-subject bias and precision, defined as the mean and standard deviation (SD) of estimation error for all time samples, were calculated for each trial. The intra-subject bias and precision in the sagittal plane can be calculated using Eq. 10 and Eq. 11; similar equations were used in the frontal and transverse plane.

9 $$\begin{aligned} error_{n}= & {} \theta _{n \mid ref} - \theta _{n \mid x} \end{aligned}$$

10 $$\begin{aligned} bias= & {} mean \left(\sum _n error \right) \end{aligned}$$

11 $$\begin{aligned} precision= & {} SD \left(\sum _n error \right) \end{aligned}$$

where $\theta$ is the orientation, *n* is the number of time sample, *ref* is the reference method, and *x* is the proposed NARX model.

Furthermore, the root mean square error (RMSE) of each subject was also calculated. Linear dependency between the NARX model and the reference was quantified by computing the Pearson’s correlation coefficients (*r*). Results of the study were reported in the following section as the means and standard deviations of the intra-subject biases, precisions, RMSEs, and correlation coefficients. Results were also compared across different categories, i. e., between the intact limb and prosthetic limb, below knee prosthesis (BK) and above knee prosthesis (AK), and the robotic foot and passive foot. Agreement between the NARX model and the reference method was evaluated in a graphical means using the Bland-Altman plots [40]. Signal processing, data analysis, and statistical evaluations of the entire dataset were performed using MATLAB (R2021a, The Mathworks Inc., Natick, MA, USA).

## Results

**Fig. 3:**
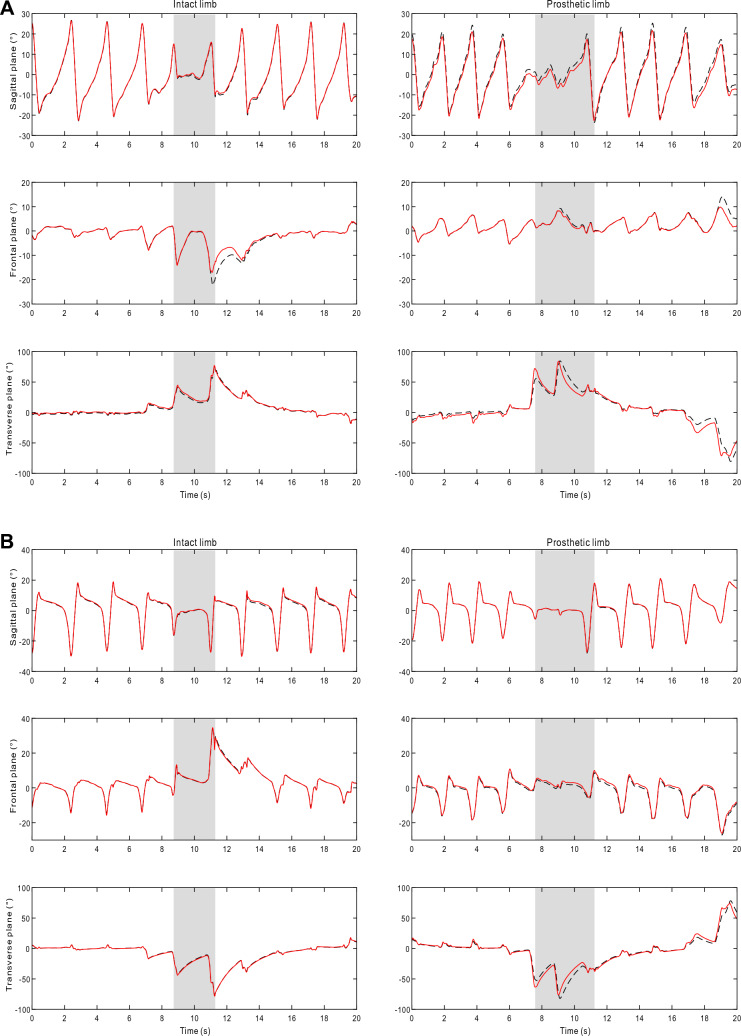
Example of typical 3D orientation for the **(A)** shank and **(B)** foot of the intact limb and prosthetic limb (with the robotic foot), computed by the proposed NARX model (red solid line) and the reference CF (dashed line) during a random 20 s walk. Gray areas denote U-turn while white areas denote level walking

Data obtained through 26 trials (13 subjects, 2 trials each subject) were analyzed. For the sake of clarity, comparisons were categorized into level walking and U-turn for each plane of movement. The turning angle, computed as the difference of azimuth angle between two successive gait events, was used as the metric that separates straight walking and U-turn [41]. A total of 1266 gait cycles were collected during level walking, of which the intact and prosthetic limbs recorded 578 and 688 gait cycles, respectively. Gait cycles of the prosthetic limb were further categorized into 484 cycles of BK and 204 cycles of AK. Moreover, level walking using the passive foot and the robotic foot comprised 364 and 324 gait cycles, respectively. A total of 308 turns were obtained during U-turn, involving 142 turns on the intact side and 166 turns on the prosthetic side. U-turn with the BK and AK consisted 112 and 54 turns, while the passive foot and the robotic foot consisted 90 and 76 turns, respectively. An example of the 3D shank and foot orientation computed by the NARX model and the CF, for a trial using the robotic foot is presented in Fig. 3. Similar profiles were found for all trials using the passive foot in the study.

Performance of the proposed NARX model in estimating sagittal shank and foot movements during level walking and U-turn is summarized in Table 3 and Table 4 as the inter-subject mean, SD, minimum (min), and maximum (max) values of bias, precision, RMSE, and correlation coefficient (*r*). In general, the foot was found to exhibit lower estimation errors during level walking, with the overall mean ± SD values of 0.02 ± 0.08° for bias, 0.35 ± 0.05° for precision, 0.36 ± 0.05° for RMSE, and 0.999 ± 0.002 for *r*. Higher accuracy was also found in the foot segment during U-turn, with a bias of 0.02 ± 0.09°, precision of 0.32 ± 0.07°, RMSE of 0.33 ± 0.07°, and *r* value of 0.999 ± 0.002.

For the estimation of shank and foot orientation in the frontal plane during level walking and U-turn (Table 5 and Table 6), lower estimation errors were obtained in the foot segment for both activities. The overall mean ± SD values of bias, precision, RMSE, and *r* for the foot during level walking were -0.01 ± 0.18°, 0.40 ± 0.14°, 0.44 ± 0.15°, and 0.996 ± 0.007, respectively. On the other hand, the foot performing U-turn recorded a bias of − 0.02 ± 0.20°, precision of 0.50 ± 0.25°, RMSE of 0.53 ± 0.25°, and *r* value of 0.997 ± 0.005.

Table 7 and Table 8 show the comparisons of shank and foot orientation estimation in the transverse plane for level walking and U-turn. The shank demonstrated better performance in level walking, with a general mean ± SD of -0.08 ± 0.27° for bias, 1.11 ± 0.21° for precision, 1.15 ± 0.19° for RMSE, and 0.993 ± 0.011 for *r*. During U-turn, the shank demonstrated a bias of -0.23 ± 0.47°, precision of 1.73 ± 0.66°, RMSE of 1.82 ± 0.61°, and *r* value of 0.998 ± 0.004.

**Fig. 4:**
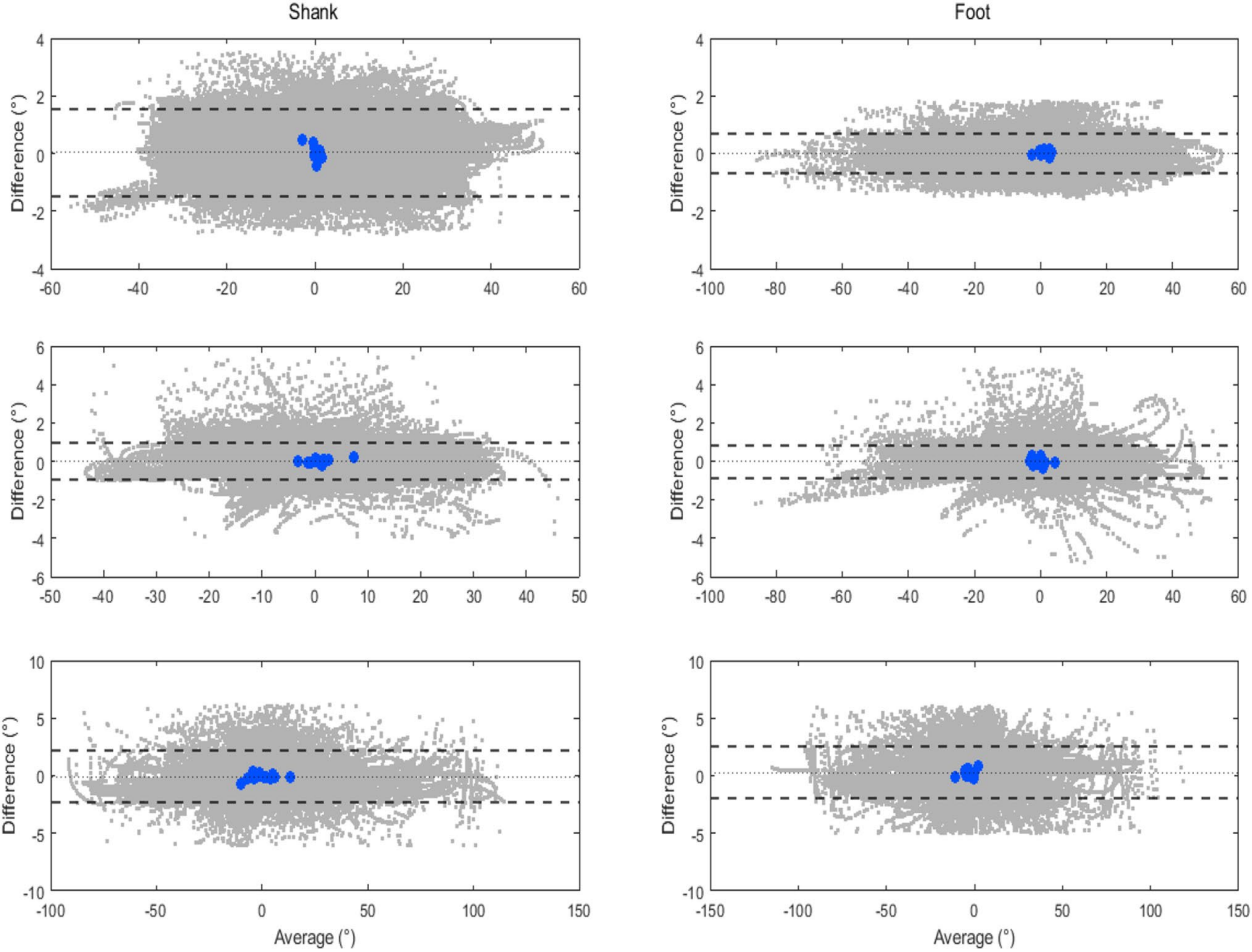
Bland-Altman plots of the proposed NARX model in the sagittal (top panel), frontal (middle panel), and transverse plane (bottom panel) for the shank and foot, during level walking. The gray dots represent measurement errors of all time samples for the trials performed by all subjects and the blue circles represent the biases of each individual across all trials. The dotted line corresponds to the mean, and the dashed lines represent the upper and lower limits of agreement (1.96 SD), respectively

**Fig. 5:**
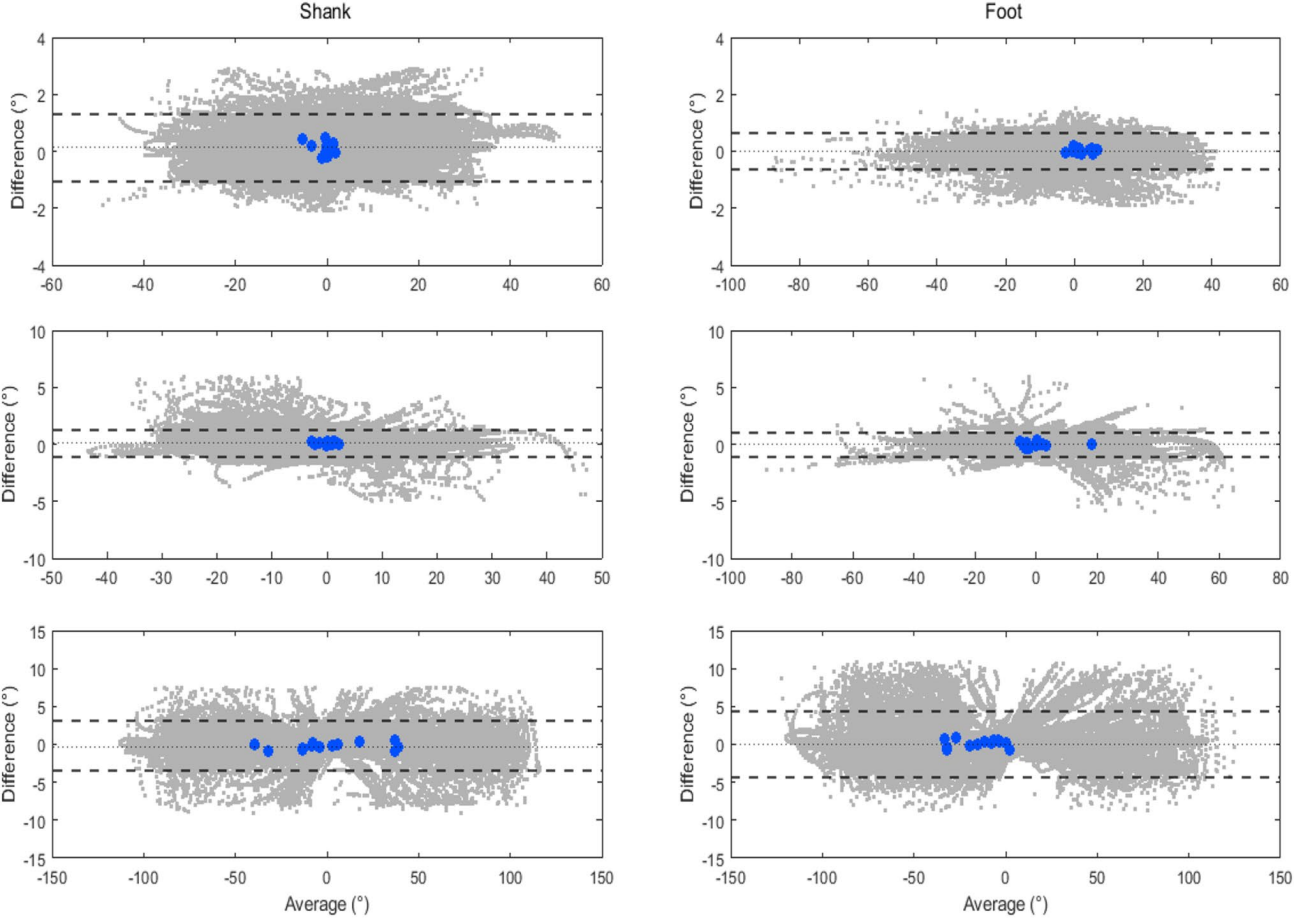
Bland-Altman plots of the proposed NARX model in the sagittal (top panel), frontal (middle panel), and transverse plane (bottom panel) for the shank and foot, during U-turn. The gray dots represent measurement errors of all time samples for the trials performed by all subjects and the blue circles represent the biases of each individual across all trials. The dotted line corresponds to the mean, and the dashed lines represent the upper and lower limits of agreement (1.96 SD), respectively

Agreement between the shank and foot orientation estimated using the proposed model and the reference method is illustrated in Fig. 4 and Fig. 5. The Bland-Altman plots include differences (i.e., errors, computed using Eq. 9) at each time sample for all subjects (represented as gray dots) and biases (i.e., mean error across all trials, computed using Eq. 10) for each subject (represented as blue circles, with a total of 13 points corresponding to 13 subjects) in all movement planes during level walking and U-turn.

**Table 3:** Inter-subject mean, SD, minimum (Min), and maximum (Max) of bias, precision, RMSE, and *r* for shank orientation in the sagittal plane during level walking and U-turn

Activity		Bias ($^\circ$)					Precision ($^\circ$)					RMSE ($^\circ$)					*r*			
		Mean	SD	Min	Max		Mean	SD	Min	Max		Mean	SD	Min	Max		Mean	SD	Min	Max
Level	Intact	0.09	0.42	− 0.47	0.97		0.64	0.22	0.30	1.05		0.74	0.28	0.32	1.29		0.998	0.002	0.995	1.000
walking	Prosthetic	0.10	0.41	− 0.70	0.91		0.62	0.23	0.33	1.22		0.73	0.24	0.39	1.22		0.993	0.020	0.927	1.000
	BK	− 0.05	0.38	− 0.70	0.37		0.61	0.17	0.33	0.82		0.69	0.22	0.39	1.04		0.999	0.001	0.996	1.000
	AK	0.14	0.22	− 0.07	0.43		0.74	0.33	0.49	1.22		0.79	0.29	0.51	1.22		0.984	0.032	0.927	1.000
	Robotic	0.22	0.58	− 0.10	1.20		0.56	0.22	0.26	0.96		0.78	0.33	0.28	1.39		0.996	0.011	0.959	1.000
	Passive	− 0.18	0.47	− 0.85	0.39		0.48	0.20	0.16	0.73		0.65	0.28	0.16	1.07		0.996	0.009	0.969	1.000
	**Overall**	**0.07**	**0.23**	**− 0.44**	**0.49**		**0.71**	**0.17**	**0.46**	**1.04**		**0.74**	**0.20**	**0.47**	**1.10**		**0.997**	**0.004**	**0.985**	**1.000**
U− turn	Intact	0.13	0.40	− 0.37	1.09		0.53	0.18	0.20	0.82		0.63	0.28	0.22	1.29		0.999	0.001	0.996	1.000
	Prosthetic	0.19	0.37	− 0.25	1.03		0.44	0.18	0.20	0.89		0.57	0.20	0.33	1.06		0.995	0.014	0.950	1.000
	BK	0.08	0.22	− 0.25	0.36		0.44	0.14	0.20	0.60		0.50	0.10	0.33	0.63		0.998	0.003	0.993	1.000
	AK	0.22	0.23	− 0.10	0.50		0.57	0.26	0.36	0.89		0.66	0.22	0.36	0.89		0.989	0.022	0.950	1.000
	Robotic	0.15	0.61	− 1.38	1.03		0.43	0.20	0.20	0.89		0.70	0.31	0.27	1.42		0.994	0.018	0.933	1.000
	Passive	− 0.03	0.33	− 0.60	0.43		0.33	0.18	0.11	0.65		0.45	0.19	0.13	0.86		0.997	0.005	0.985	1.000
	**Overall**	**0.12**	**0.21**	**− 0.22**	**0.46**		**0.56**	**0.13**	**0.36**	**0.79**		**0.60**	**0.15**	**0.37**	**0.92**		**0.997**	**0.004**	**0.984**	**1.000**

**Table 4:** Inter− subject mean, SD, minimum (Min), and maximum (Max) of bias, precision, RMSE, and *r* for foot orientation in the sagittal plane during level walking and U− turn

Activity		Bias ($^\circ$)					Precision ($^\circ$)					RMSE ($^\circ$)					*r*			
		Mean	SD	Min	Max		Mean	SD	Min	Max		Mean	SD	Min	Max		Mean	SD	Min	Max
Level	Intact	0.07	0.12	− 0.09	0.29		0.35	0.14	0.10	0.54		0.37	0.14	0.15	0.55		0.999	0.001	0.998	1.000
walking	Prosthetic	− 0.03	0.15	− 0.09	0.29		0.29	0.14	0.06	0.52		0.33	0.14	0.15	0.64		0.999	0.004	0.985	1.000
	BK	− 0.08	0.16	− 0.37	0.07		0.31	0.14	0.13	0.52		0.35	0.15	0.15	0.64		1.000	0.000	0.999	1.000
	AK	0.07	0.11	− 0.09	0.22		0.29	0.13	0.17	0.46		0.32	0.11	0.20	0.48		0.997	0.006	0.985	1.000
	Robotic	− 0.01	0.17	− 0.42	0.19		0.24	0.15	0.06	0.57		0.28	0.17	0.11	0.71		0.999	0.004	0.985	1.000
	Passive	− 0.08	0.22	− 0.41	0.29		0.35	0.13	0.15	0.54		0.41	0.12	0.23	0.65		0.998	0.006	0.978	1.000
	**Overall**	**0.02**	**0.08**	**− 0.15**	**0.16**		**0.35**	**0.05**	**0.26**	**0.41**		**0.36**	**0.05**	**0.26**	**0.42**		**0.999**	**0.002**	**0.993**	**1.000**
U− turn	Intact	0.07	0.17	− 0.28	0.38		0.30	0.14	0.07	0.52		0.35	0.13	0.15	0.52		0.999	0.001	0.998	1.000
	Prosthetic	− 0.02	0.21	− 0.53	0.28		0.25	0.11	0.10	0.46		0.31	0.15	0.14	0.70		0.999	0.003	0.988	1.000
	BK	− 0.12	0.18	− 0.53	0.10		0.25	0.12	0.10	0.46		0.30	0.18	0.14	0.70		1.000	0.001	0.998	1.000
	AK	0.15	0.15	− 0.03	0.34		0.26	0.10	0.14	0.41		0.34	0.08	0.23	0.42		0.997	0.005	0.988	1.000
	Robotic	− 0.03	0.30	− 0.96	0.27		0.20	0.11	0.06	0.42		0.28	0.25	0.07	1.02		0.999	0.004	0.987	1.000
	Passive	− 0.06	0.31	− 0.64	0.41		0.26	0.13	0.12	0.56		0.39	0.18	0.25	0.85		0.997	0.008	0.992	1.000
	**Overall**	**0.02**	**0.09**	**− 0.07**	**0.21**		**0.32**	**0.07**	**0.21**	**0.44**		**0.33**	**0.07**	**0.22**	**0.46**		**0.999**	**0.002**	**0.992**	**1.000**

**Table 5:** Inter− subject mean, SD, minimum (Min), and maximum (Max) of bias, precision, RMSE, and *r* for shank orientation in the frontal plane during level walking and U-turn

Activity		Bias ($^\circ$)					Precision ($^\circ$)					RMSE ($^\circ$)					*r*			
		Mean	SD	Min	Max		Mean	SD	Min	Max		Mean	SD	Min	Max		Mean	SD	Min	Max
Level	Intact	0.08	0.21	− 0.18	0.37		0.50	0.13	0.30	0.75		0.55	0.13	0.35	0.77		0.996	0.003	0.990	1.000
walking	Prosthetic	− 0.03	0.19	− 0.32	0.28		0.37	0.17	0.08	0.61		0.41	0.19	0.08	0.67		0.997	0.003	0.989	1.000
	BK	− 0.03	0.19	− 0.32	0.24		0.36	0.17	0.10	0.52		0.40	0.18	0.11	0.57		0.998	0.003	0.992	1.000
	AK	− 0.03	0.21	− 0.30	0.28		0.43	0.12	0.26	0.61		0.46	0.15	0.26	0.67		0.996	0.004	0.989	1.000
	Robotic	0.01	0.14	− 0.22	0.32		0.23	0.15	0.08	0.50		0.26	0.16	0.08	0.59		0.999	0.002	0.992	1.000
	Passive	− 0.05	0.44	− 0.65	1.10		0.46	0.23	0.11	0.87		0.57	0.35	0.11	1.40		0.993	0.017	0.986	1.000
	**Overall**	**0.03**	**0.11**	**− 0.20**	**0.23**		**0.47**	**0.13**	**0.15**	**0.68**		**0.48**	**0.13**	**0.19**	**0.68**		**0.997**	**0.003**	**0.986**	**1.000**
U− turn	Intact	0.24	0.24	− 0.12	0.59		0.60	0.34	0.20	1.16		0.67	0.37	0.20	1.23		0.996	0.005	0.985	1.000
	Prosthetic	− 0.04	0.16	− 0.32	0.34		0.50	0.23	0.12	1.04		0.52	0.24	0.12	1.04		0.994	0.010	0.963	1.000
	BK	− 0.08	0.15	− 0.32	0.07		0.45	0.16	0.15	0.71		0.48	0.17	0.18	0.75		0.998	0.002	0.994	1.000
	AK	0.05	0.17	− 0.08	0.34		0.59	0.30	0.20	1.04		0.61	0.30	0.21	1.04		0.989	0.015	0.963	1.000
	Robotic	− 0.04	0.14	− 0.37	0.10		0.32	0.20	0.07	0.61		0.34	0.21	0.09	0.69		0.997	0.004	0.988	1.000
	Passive	− 0.07	0.37	− 0.87	0.57		0.57	0.42	0.19	1.66		0.68	0.42	0.20	1.67		0.988	0.027	0.906	1.000
	**Overall**	**0.08**	**0.11**	**− 0.09**	**0.25**		**0.58**	**0.25**	**0.15**	**0.96**		**0.59**	**0.25**	**0.18**	**0.99**		**0.996**	**0.005**	**0.982**	**1.000**

**Table 6:** Inter-subject mean, SD, minimum (Min), and maximum (Max) of bias, precision, RMSE, and *r* for foot orientation in the frontal plane during level walking and U-turn

Activity		Bias ($^\circ$)					Precision ($^\circ$)					RMSE ($^\circ$)					*r*			
		Mean	SD	Min	Max		Mean	SD	Min	Max		Mean	SD	Min	Max		Mean	SD	Min	Max
Level	Intact	− 0.05	0.21	− 0.40	0.32		0.35	0.15	0.14	0.64		0.40	0.15	0.19	0.65		0.996	0.008	0.972	1.000
walking	Prosthetic	0.04	0.25	− 0.37	0.61		0.42	0.18	0.25	0.78		0.47	0.22	0.27	0.99		0.995	0.008	0.972	1.000
	BK	0.10	0.28	− 0.37	0.61		0.48	0.20	0.26	0.78		0.53	0.26	0.27	0.99		0.997	0.003	0.989	0.999
	AK	− 0.07	0.17	− 0.28	0.10		0.30	0.04	0.25	0.35		0.34	0.05	0.27	0.38		0.993	0.012	0.972	1.000
	Robotic	0.08	0.25	− 0.49	0.49		0.27	0.16	0.03	0.60		0.35	0.20	0.03	0.76		0.999	0.001	0.997	1.000
	Passive	− 0.02	0.39	− 0.50	0.82		0.47	0.31	0.11	1.10		0.57	0.35	0.16	1.37		0.993	0.015	0.948	1.000
	**Overall**	**− 0.01**	**0.18**	**− 0.33**	**0.31**		**0.40**	**0.14**	**0.23**	**0.72**		**0.44**	**0.15**	**0.24**	**0.76**		**0.996**	**0.007**	**0.973**	**1.000**
U− turn	Intact	− 0.13	0.24	− 0.45	0.21		0.44	0.25	0.10	0.86		0.51	0.27	0.21	0.97		0.998	0.003	0.989	1.000
	Prosthetic	0.07	0.29	− 0.30	0.72		0.50	0.25	0.20	1.05		0.57	0.27	0.25	1.09		0.995	0.009	0.969	0.999
	BK	0.14	0.31	− 0.23	0.72		0.55	0.30	0.20	1.05		0.63	0.33	0.25	1.09		0.997	0.004	0.988	0.999
	AK	− 0.07	0.21	− 0.30	0.13		0.41	0.13	0.31	0.61		0.45	0.15	0.31	0.68		0.993	0.014	0.969	0.999
	Robotic	0.09	0.37	− 0.70	0.64		0.32	0.19	0.03	0.78		0.45	0.26	0.08	0.93		0.999	0.002	0.991	1.000
	Passive	0.07	0.43	− 0.61	0.81		0.51	0.39	0.13	1.36		0.65	0.41	0.15	1.37		0.990	0.017	0.943	1.000
	**Overall**	**− 0.02**	**0.20**	**− 0.33**	**0.38**		**0.50**	**0.25**	**0.20**	**1.04**		**0.53**	**0.25**	**0.25**	**1.04**		**0.997**	**0.005**	**0.982**	**1.000**

**Table 7:** Inter− subject mean, SD, minimum (Min), and maximum (Max) of bias, precision, RMSE, and *r* for shank orientation in the transverse plane during level walking and U− turn

Activity		Bias ($^\circ$)					Precision ($^\circ$)					RMSE ($^\circ$)					*r*			
		Mean	SD	Min	Max		Mean	SD	Min	Max		Mean	SD	Min	Max		Mean	SD	Min	Max
Level	Intact	0.01	0.32	− 0.38	0.56		1.09	0.27	0.68	1.42		1.13	0.28	0.69	1.45		0.994	0.010	0.963	0.999
walking	Prosthetic	− 0.08	0.38	− 0.68	0.43		1.10	0.30	0.54	1.60		1.16	0.30	0.55	1.60		0.991	0.012	0.961	0.999
	BK	− 0.24	0.39	− 0.68	0.24		1.11	0.26	0.76	1.52		1.20	0.24	0.82	1.54		0.997	0.002	0.993	0.999
	AK	0.22	0.13	0.05	0.43		1.18	0.26	0.95	1.60		1.20	0.26	0.97	1.60		0.983	0.016	0.961	0.997
	Robotic	− 0.17	0.51	− 1.03	0.53		0.99	0.34	0.54	1.54		1.12	0.32	0.55	1.58		0.992	0.013	0.955	1.000
	Passive	0.06	0.44	− 0.65	0.59		1.32	0.44	0.57	2.06		1.40	0.43	0.70	2.07		0.990	0.011	0.961	0.999
	**Overall**	**− 0.08**	**0.27**	**− 0.68**	**0.39**		**1.11**	**0.21**	**0.74**	**1.38**		**1.15**	**0.19**	**0.75**	**1.43**		**0.993**	**0.011**	**0.962**	**0.999**
U− turn	Intact	− 0.20	0.54	− 1.23	0.73		1.65	0.61	0.85	2.69		1.73	0.63	0.88	2.87		0.998	0.003	0.988	1.000
	Prosthetic	− 0.21	0.79	− 1.45	1.22		1.91	0.89	0.68	3.18		2.07	0.87	0.81	3.27		0.997	0.004	0.983	1.000
	BK	− 0.14	0.78	− 0.89	1.22		1.93	0.95	0.68	3.18		2.09	0.90	0.81	3.27		0.999	0.007	0.983	0.999
	AK	− 0.35	0.86	− 1.45	0.66		1.98	0.76	1.34	2.85		2.13	0.81	1.47	3.11		0.995	0.007	0.983	0.999
	Robotic	− 0.41	0.99	− 1.97	1.08		1.64	1.03	0.58	3.15		2.01	0.89	0.89	3.19		0.998	0.001	0.996	1.000
	Passive	0.06	0.82	− 1.03	1.55		2.25	0.97	0.51	3.19		2.38	0.97	0.59	3.35		0.994	0.007	0.978	1.000
	**Overall**	**− 0.23**	**0.47**	**− 0.97**	**0.61**		**1.73**	**0.66**	**0.68**	**2.80**		**1.82**	**0.61**	**0.84**	**2.83**		**0.998**	**0.004**	**0.985**	**1.000**

**Table 8:** Inter-subject mean, SD, minimum (Min), and maximum (Max) of bias, precision, RMSE, and *r* for foot orientation in the transverse plane during level walking and U-turn

Activity		Bias ($^\circ$)					Precision ($^\circ$)					RMSE ($^\circ$)					*r*			
		Mean	SD	Min	Max		Mean	SD	Min	Max		Mean	SD	Min	Max		Mean	SD	Min	Max
Level	Intact	0.09	0.42	− 0.72	0.66		0.92	0.25	0.56	1.33		1.01	0.27	0.57	1.33		0.992	0.019	0.933	1.000
walking	Prosthetic	0.43	0.35	− 0.08	1.07		1.27	0.35	0.59	1.70		1.38	0.39	0.60	1.85		0.988	0.017	0.942	1.000
	BK	0.43	0.41	− 0.08	1.07		1.17	0.39	0.59	1.70		1.29	0.45	0.60	1.85		0.997	0.002	0.993	1.000
	AK	0.48	0.31	0.09	0.79		1.44	0.25	1.05	1.65		1.54	0.30	1.10	1.83		0.976	0.023	0.942	0.992
	Robotic	0.42	0.44	− 0.32	1.12		1.09	0.40	0.39	1.61		1.25	0.40	0.59	1.86		0.989	0.015	0.946	1.000
	Passive	0.47	0.78	− 1.10	2.03		1.35	0.47	0.59	2.05		1.59	0.55	0.60	2.27		0.982	0.028	0.903	1.000
	**Overall**	**0.24**	**0.32**	**− 0.21**	**0.84**		**1.11**	**0.27**	**0.59**	**1.51**		**1.17**	**0.29**	**0.60**	**1.51**		**0.992**	**0.016**	**0.941**	**1.000**
U− turn	Intact	− 0.18	0.49	− 1.17	0.46		1.87	0.75	0.62	3.18		1.95	0.70	0.76	3.19		0.997	0.005	0.981	1.000
	Prosthetic	0.70	0.84	− 0.80	2.44		2.75	0.98	0.73	4.08		2.92	1.05	0.79	4.28		0.994	0.007	0.978	0.999
	BK	0.44	0.74	− 0.80	1.31		2.73	1.13	0.73	4.08		2.83	1.17	0.79	4.28		0.997	0.002	0.994	0.999
	AK	1.09	0.89	0.19	2.44		2.77	0.80	1.89	3.44		3.05	0.93	1.94	4.04		0.991	0.008	0.978	0.998
	Robotic	0.62	0.87	− 0.43	2.72		2.43	1.23	0.58	3.99		2.63	1.25	0.85	4.04		0.996	0.004	0.984	1.000
	Passive	0.88	1.15	− 1.83	2.22		2.93	1.10	0.71	4.07		3.25	1.10	0.73	4.48		0.988	0.017	0.955	0.999
	**Overall**	**0.12**	**0.48**	**− 0.78**	**0.81**		**2.25**	**0.64**	**0.73**	**3.10**		**2.30**	**0.63**	**0.79**	**3.10**		**0.996**	**0.006**	**0.976**	**0.999**

## Discussion

This study proposed a neural network-based method to estimate the shank and foot orientation of lower limb amputees using body-worn inertial sensors. First, the proposed NARX model was trained using the sagittal plane data originated from a healthy individual, with the foot angular velocity and foot orientation computed by the CF, as the input and target output. The trained model was then applied to compute 3D segmental orientation of the lower limb amputees using the gyroscope signals as the input. In this study, the CF was selected as the reference method due to its well-known simplicity and computational efficacy [42, 43]. The feasibility of CF in estimating body segment orientation had also been validated in Tham *et. al.* [33]. Performance of the proposed method was assessed as comparisons with the reference method, to validate the capability of the NARX model in resembling the CF in computing segment orientation using only the gyroscope signals.

Generally, estimation biases produced in the foot were slightly lower compared to the shank for the sagittal and frontal planes. Better orientation prediction in the foot segment was the outcome of using the NARX model trained with the foot kinematics. On the other hand, biases, precisions, and RMSEs produced in the transverse plane were the highest among the three movement planes, for both the shank and foot. Influenced by ferromagnetic interference of the clinical surroundings, performance of the magnetometer in the transverse plane was negatively affected, thus reducing the accuracy of orientation estimates using the CF (i.e., reference method). This explains the greater difference between the reference and the proposed model in which the NARX model was not affected in the transverse plane. Furthermore, higher estimation errors in U-turn observed across all movement planes and lower limb categories were the effect of higher translational acceleration experienced by the lower limb segments during a rapid change in moving direction.

Despite the differences between lower limb segment and ambulation activities (Table 3 - Table 8), the NARX model generally estimated the shank and foot orientation with high accuracy. The small biases of less than 0.3° in the sagittal and frontal plane, and less than 1.5° in the transverse plane, correspond to the variation smaller than 0.5% (sagittal and frontal plane) and 0.75% (transverse plane) of the range of movement in the respective planes. Existing work of prosthetic gait assessment using the NARX neural network is not available, however, the variations of measurement errors in this study are comparable to the previous studies in other applications, involving comparisons to the CF [42, 44]. The results were further supported by the extremely high *r* values for the sagittal (0.984 to 1), frontal (0.988 to 0.999), and the transverse (0.982 to 0.997) planes. Furthermore, the Bland-Altman plots (Fig. 4 and Fig. 5) show that the majority of biases between the NARX and the reference method, for every time sample throughout the trials, were within the limits of agreement, except for some minimal outliers where the biases were within the acceptable range.

The minor differences between all groups of comparisons (Table 3 - Table 8) indicate the ability of the proposed NARX model to replicate the properties of the CF in orientation estimation of the amputee’s gait in all movement planes, although the model was trained using data in the sagittal plane of a healthy individual. Differences were not observed in the bias range of the prosthetic limb compared to the intact limb, regardless of the level of amputation and the type of prosthesis used during ambulation. These show generalization properties of the proposed NARX model in orientation estimation, which demonstrate the potential of a plug-and-play technique that is readily available to accommodate measurements of any gait types in the clinical environment without the hassle of retraining the network model.

The results also show the advantages of the NARX neural network over other types of neural networks. Possessing the properties of generalization, the general regression neural network (GRNN) is one of the common techniques used in biomechanics for movement recognition and prediction [45–47]. However, accuracy of the GRNN was shown to be low with small datasets being included to the models [48–50]. A sufficiently large dataset that includes vast variety of gait characteristics was suggested to train a GRNN model in order to reduce prediction errors in individuals of different populations [48]. Successive GRNN models developed on the basis of GRNN could also be the alternatives to improve the performance and accuracy [49–51]. On the other hand, the proposed NARX model, with its minimal architecture, exhibited high accuracy in estimating segmental orientation given the diversity of the subject groups. This verified the ability of the NARX model to generate accurate predictions of human gait which is categorized as complex nonlinear time series data [31, 33, 52, 53].

Moreover, using only gyroscopes, the proposed NARX model is capable of computing 3D segmental orientation of the amputees effectively while avoiding measurement errors arising from the gravitational acceleration and magnetic field interference. This enables a more accessible, reliable, and accurate prosthetic gait assessment especially in the clinical applications. The proposed method can also be used in long term observation and monitoring for people with lower limb amputation by embedding IMUs in the prosthesis and its components. The robust technique can also provide reliable feedback to robotic prosthesis for a better control that ease mobility in daily life.

Nevertheless, there are some limitations of this study that are worth noting. First, the proposed model was compared to the CF as the reference instead of the optical motion capture system which is often considered as the gold standard. This was due to the unavailability of the motion capture cameras at the location of trials. Setup of the motion capture system is commonly a huge challenge, as the system requires high cost with dedicated space and personnel, which is frequently not favorable in clinical environments [54–57]. Despite of that, the validity of CF in estimating 3D body segment orientation has been proven in previous study [33] and thus feasible to be selected as the reference of the present study. Second, trials of the study involved short walking distance that might not be sufficient to show the continuous performance of the proposed method in orientation estimation over prolonged measurement course. This could be improved in future studies quantifying amputees’ gait in long-distance and real-life environments with more segmental orientation and joint angles to provide a comprehensive outcome measure in quantitative prosthetic gait assessment.

## Conclusion

The proposed NARX network model demonstrated high accuracy in the estimation of 3D lower limb segment orientation of the lower limb amputees. Compared to the CF, overall inter-subject RMSEs during level walking were 0.74 ± 0.20°, 0.48 ± 0.13°, and 1.15 ± 0.19° for the shank and 0.36 ± 0.05°, 0.44 ± 0.15°, and 1.17 ± 0.29° for the foot, in the sagittal, frontal, and transverse planes, respectively. U-turn yielded the overall RMSEs of 0.60 ± 0.15°, 0.59 ± 0.25°, and 1.82 ± 0.61° for the shank and 0.33 ± 0.07°, 0.53 ± 0.25°, and 2.30 ± 0.63° for the foot, in the sagittal, frontal, and transverse planes, respectively. Resembling the properties of CF, the proposed method is capable of estimating orientation without incorporating drift errors and ferromagnetic interference to the model. Future work should focus on long-term performance evaluation of the NARX model to investigate the potential of the model as a quantitative measure to clinical assessment and monitoring of the prosthetic gait.

## Data Availability

Data sharing is not applicable to this article.
